# A virus-specific monocyte inflammatory phenotype is induced by SARS-CoV-2 at the immune–epithelial interface

**DOI:** 10.1073/pnas.2116853118

**Published:** 2021-12-28

**Authors:** Juliette Leon, Daniel A. Michelson, Judith Olejnik, Kaitavjeet Chowdhary, Hyung Suk Oh, Adam J. Hume, Silvia Galván-Peña, Yangyang Zhu, Felicia Chen, Brinda Vijaykumar, Liang Yang, Elena Crestani, Lael M. Yonker, David M. Knipe, Elke Mühlberger, Christophe Benoist

**Affiliations:** ^a^Department of Immunology, Blavatnik Institute, Harvard Medical School, Boston, MA 02115;; ^b^INSERM UMR 1163, University of Paris, Institut Imagine, 75015 Paris, France;; ^c^Department of Microbiology, Boston University School of Medicine, Boston, MA 02118;; ^d^National Emerging Infectious Diseases Laboratories, Boston University, Boston, MA 02215;; ^e^Department of Microbiology, Blavatnik Institute, Harvard Medical School, Boston, MA 02115;; ^f^Division of Immunology, Boston Children's Hospital, Harvard Medical School, Boston, MA 02115;; ^g^Department of Pediatrics, Harvard Medical School, Boston, MA 02115;; ^h^Department of Pediatrics, Massachusetts General Hospital, Boston, MA 02114

**Keywords:** COVID-19, cytokine storm, interferon

## Abstract

Infection by severe acute respiratory syndrome coronavirus-2 (SARS-CoV-2) provokes a potentially fatal pneumonia with multiorgan failure, and high systemic inflammation. To gain mechanistic insight and ferret out the root of this immune dysregulation, we modeled, by in vitro coculture, the interactions between infected epithelial cells and immunocytes. A strong response was induced in monocytes and B cells, with a SARS-CoV-2–specific inflammatory gene cluster distinct from that seen in influenza A or Ebola virus-infected cocultures, and which reproduced deviations reported in blood or lung myeloid cells from COVID-19 patients. A substantial fraction of the effect could be reproduced after individual transfection of several SARS-CoV-2 proteins (Spike and some nonstructural proteins), mediated by soluble factors, but not via transcriptional induction. This response was greatly muted in monocytes from healthy children, perhaps a clue to the age dependency of COVID-19. These results suggest that the inflammatory malfunction in COVID-19 is rooted in the earliest perturbations that SARS-CoV-2 induces in epithelia.

## Significance

By modeling in vitro the cross-talk between epithelial and immune cells, this work provides possible origins for the profound inflammatory perturbations that are a hallmark of COVID-19, and the relative protection of children from severe disease. The initial interaction between immune cells and epithelial cells infected with SARS-CoV-2, or transduced to express the proteins the virus encodes, elicits a specific response, not observed with other pathogenic viruses, that presages perturbations seen in patients with severe COVID-19. Thus, the severe manifestations of COVID-19 may be rooted in the very first response that it elicits from immunocytes.

Viral infections induce varied innate and inflammatory responses in the host. These responses help to control the viruses, but in some cases can become far more deleterious than the virus itself (1). Infection with severe acute respiratory syndrome coronavirus-2 [SARS-CoV-2 (CoV2)], the cause of the current COVID-19 pandemic, leads to an upper respiratory tract infection which, if not controlled by the innate and adaptive immune responses, can evolve into a lethal pneumonia. CoV2 infection is remarkable in its clinical heterogeneity, ranging from asymptomatic to fatal (2), and several clinical characteristics demarcate the pathology associated with CoV2, when compared with other respiratory pathogens such as influenza A virus (IAV). First, critical COVID-19 is associated with multiorgan failure beyond the lungs and a concomitant severe vasculopathy (3–5). Second, bacterial coinfection, a common complication in IAV infections (6, 7), is rarely found in COVID-19, yet COVID-19 nonetheless adopts clinical aspects of bacterial sepsis (8), with an overeffusive production of inflammatory cytokines (reviewed in ref. 9). Finally, an important feature of COVID-19 is that children are usually spared from severe disease, showing asymptomatic or milder disease at the acute phase (10–13), even though viral loads are similar to adults (14). Such an age imbalance is not seen in IAV infections.

Many studies have aimed to understand the molecular and immunological factors that drive these clinical phenotypes (15–19). In severe COVID-19, profound alterations of the immune system have been described in myeloid cells (20, 21), along with impaired interferon (IFN) responses (22–24), impaired T cell functions (25–28), production of autoantibodies (29), and high circulating levels of inflammatory cytokines (17, 24, 30). It is not obvious how to disentangle which of these manifestations causally partake in severe pathogenesis and which are only bystander markers of the strong inflammation. Direct pathogenicity from virus-induced damage is unlikely to be a driver, as high viral loads can exist early in asymptomatic or mild disease (31, 32), pointing to a determining role of host factors. Abnormalities in the type I IFN pathway, resulting from genetic alterations (33) or from IFN-neutralizing autoantibodies (34–37), clearly have a causative or amplifying role in COVID-19, plausibly, by allowing the virus to replicate unchecked during the early phases of infection, before adaptive immune defenses can be recruited. However, the response to CoV2 involves many cellular and molecular players, and it seems likely that additional pathways beyond type I IFN underlie both resistance and pathology. More generally, the question can be framed as understanding why the newly emerging coronaviruses, including Middle East respiratory syndrome and SARS-CoV-1, are so pathogenic, while others that have coevolved with humans are not. A plausible virologic explanation is that their molecular structures are mostly novel to human immune systems, as the H1N1 IAV variant was during the 1918 influenza pandemic, such that toxicity derives from immunologic novelty. Another hypothesis, not mutually exclusive, is that these highly pathogenic coronaviruses are equipped to perturb immune responses, perturbations which, in turn, drive severe immunopathology. Coronaviruses have large genomes, encoding many nonstructural proteins, some of which are thought to have immune-modulating capabilities (38–40). They thus have the genetic leeway to evolve such strategies, their attempts at immune evasion potentially promoting particularly deleterious immunopathology.

To better understand the root factors leading to immune dysregulation in COVID-19, we designed an in vitro coculture system in which immunocytes were exposed to epithelial cells infected with CoV2, then profiled by transcriptomics and flow cytometry. Epithelial CoV2 infection induced a strong, mixed inflammatory response in cocultured monocytes resembling that of blood monocytes from COVID-19 patients. A large component of this response was not observed with two severe human pathogens used as comparators, IAV and Ebola virus (EBOV), and this response was strikingly muted in monocytes from children. Together, these results suggest that CoV2-infected epithelial cells elicit an early and specific proinflammatory response in monocytes, which may explain the severity of COVID-19.

## Results

### In Vitro CoV2 Epithelial–Immune Coculture Induces a Mixed Inflammatory Response in Monocytes and B Cells.

**Fig. 1.**
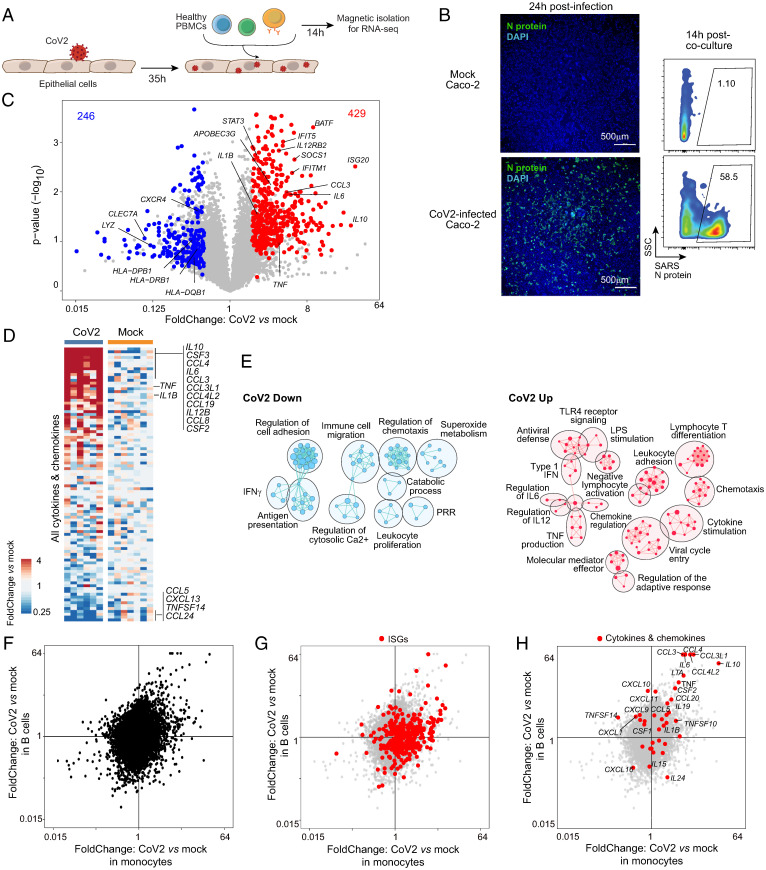
CoV2 epithelial–immune coculture triggers a mixed inflammatory response in immunocytes. (*A*) Experimental approach. Caco-2 monolayer cultures were infected with CoV2 for 2 h. Thirty-five hours postinfection, the monolayer was washed twice, and PBMCs from HDs were added directly to the cultures. These were harvested 14 h later, and subpopulations (CD14+ monocytes and CD19+ B cells) were magnetically purified for population RNAseq. (*B*) Infection rate in Caco-2 cells assessed by immunofluorescence with an anti-CoV2 N antibody: displayed by microscopy at 1 d postinfection (*Upper*, 4×, N antibody + anti-rabbit-AF488 + DAPI) and by flow cytometry at the time of harvesting PBMCs (*Lower*, ∼48 h postinfection). (*C*) FC vs. *P* value (volcano) plot of gene expression in monocytes cocultured with CoV2-infected Caco-2 compared to monocytes cocultured with uninfected Caco-2 (mock). Genes from CoV2-up signature (red) and CoV2-down signature (blue) are highlighted. (*D*) Heatmap of the expression of cytokine transcripts in CoV2 versus Mock condition (as ratio to mean of mock for each condition and each experiment). (*E*) Gene ontology analyses of CoV2-up (*Right*, red) and Cov2-down (*Left*, blue) signatures displayed as an enrichment map. Pathways are shown as circles (nodes) that are connected with lines (edges) if the pathways share many genes. Size of the node is proportional to the number of genes included in this pathway. (*F*–*H*) FC–FC plots comparing the response in monocytes (*x* axis) relative to B cells (*y* axis) in the context of CoV2 Caco-2 infection, without highlight (*F*) or highlighted with IFN-stimulated genes (ISGs) (*G*) or with cytokine and chemokine transcripts (*H*).

To assess whether and how immunocytes are triggered by CoV2-infected cells, we established a coculture model in which ex vivo blood immunocytes were placed in direct contact with virus-infected epithelial cells (Fig. 1*A*). Because primary lung epithelial cells are difficult to expand and manipulate in such conditions, we chose, as a surrogate epithelial cell, the human colorectal adenocarcinoma cell line Caco-2, which is permissive for CoV2 infection (41) and DNA transfection. Under our infection conditions, CoV2 nucleocapsid (N) expression was detected in ∼50% of Caco-2 cells by flow cytometry and immunofluorescence (Fig. 1*B*). Thirty-five hours after CoV2 infection of the Caco-2 monolayer, unbound virus was removed, and peripheral blood mononuclear cells (PBMCs) from healthy donors (HDs) were added to the cultures. These were harvested 14 h later, and subpopulations were magnetically purified for transcriptome profiling by RNA sequencing (RNAseq) (SI Appendix, Fig. S1*A*). In good part because of the experimental requirements of BSL4 biocontainment (e.g., lysates had to be heat treated for biosafety), RNAseq data quality was lower than customary. Rather than the usual statistical tests, identification of differentially expressed genes (DEGs) relied on the convergence of two independent experiments, a third experiment being used for validation (see *Materials and Methods*).

We focused the analysis on CD14^+^ monocytes and B cells, which show perturbed transcriptomes in COVID-19 patients and are both frontline sensors of infection. In purified monocytes, a robust response was observed, with at least 675 DEGs (Fig. 1*C*, which displays transcripts of the reproducible DEGs, hereafter “CoV2 signature,” and Dataset S1**)**. Immediately apparent were the induction of major proinflammatory cytokines and chemokines (*IL1B*, *IL6*, *TNF*, *CCL3*, and *CCL4*) and a substantial number of antiviral IFN stimulated genes (ISG; e.g., *IFIT5* and *ISG20*). Conversely, MHC class-II genes were significantly down-regulated. Closer examination of cytokine- and chemokine-encoding genes revealed *IL10* as the most induced cytokine transcript, along with the main proinflammatory trio (*IL6*, *IL1B*, and *TNF*; Fig. 1*D*). As analyzed further below, several of these traits evoked transcriptional changes in immunocytes of COVID-19 patients (15, 19). Gene ontology analysis of these gene sets (Fig. 1*E* and Dataset S2) revealed a complex set of functions: cytokines, innate signaling pathways, cell mobility and adherence, and antigen presentation, suggesting that exposure to CoV2-infected cells induces profound changes in monocyte physiology. In contrast, the direct transcriptomic effect of CoV2 in Caco-2 cells was very mild (see below), with none of the changes detected in monocytes.

Analysis of B cells from the same cultures also displayed numerous changes in this setting (*SI Appendix*, Fig. S1*B*). This response partially coincided with that of monocytes, but also included some components preferential or unique to either cell-type (Fig. 1*F*). Some ISGs were induced in both, although induction of the antiviral response was strongest in monocytes (Fig. 1*G*). Surprisingly, the cytokines and chemokines most strongly induced in monocytes were also induced in B cells (Fig. 1*H*). Thus, the effects of CoV2 infection on neighboring cells were apparent in several cell types.

### CoV2 Induces a Stronger Proinflammatory Response Compared to IAV and EBOV.

**Fig. 2.**
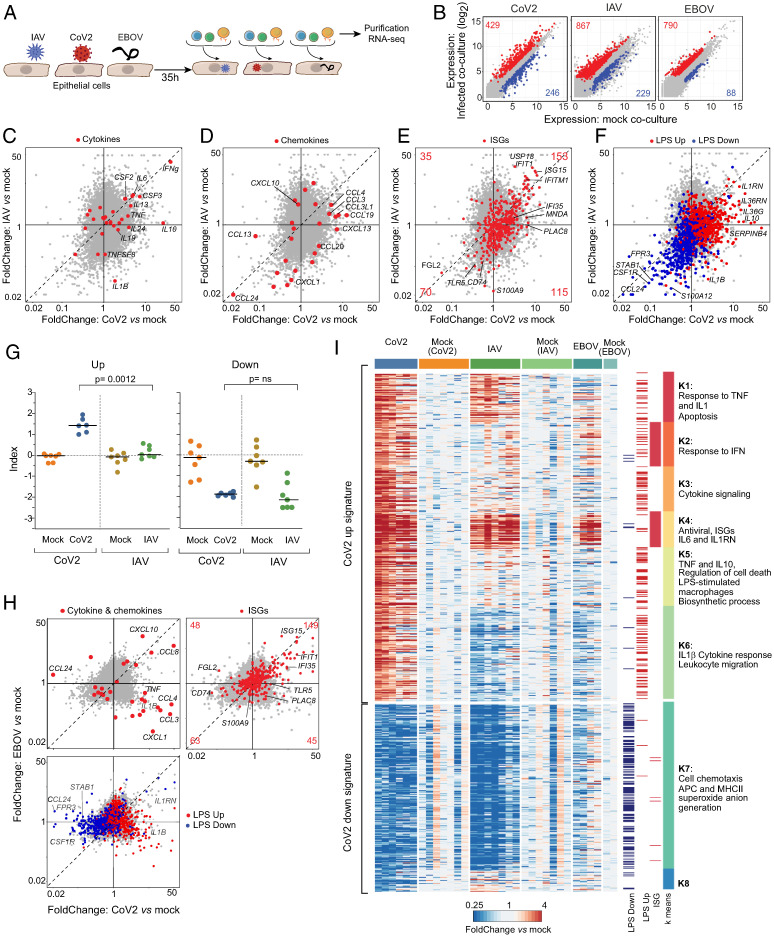
CoV2 induces a highly proinflammatory response compared to IAV and EBOV. (*A*) Experimental approach. Caco-2 monolayer cultures were infected with CoV2, IAV, or EBOV for 1, 2, and 1 h, respectively. Thirty-five hours postinfection, the monolayer was washed twice, and PBMCs from HDs were added directly to the cultures. CD14^+^ monocytes and B cells were magnetically purified 14 h later for population RNAseq. (*B*) Expression–expression plots for CoV2, IAV, and EBOV cocultured monocytes vs. their respective mock infections. Up-regulated (red) and down-regulated (blue) genes are highlighted, with numbers corresponding to the CoV2 signature as defined in Fig. 1 (CoV2), or genes with a FC > 2, *P* < 0.01 (IAV, EBOV). (*C*–*F*) FC–FC plots comparing the monocyte response to CoV2 relative to IAV, highlighted with cytokines (*C*), chemokines (*D*), IFN-stimulated genes (ISGs) (*E*), and LPS up-regulated and down-regulated gene signatures (*F*). (*G*) CoV2 up-regulated and down-regulated gene indices in CoV2, IAV, or mock-infected conditions. *P* values were calculated using the Mann–Whitney test. ns, not significant. (*H*) FC–FC plots comparing the response to CoV2 relative to EBOV, highlighted with cytokines and chemokines (*Upper Left*), IFN-stimulated genes (ISGs) (*Upper Right*), and the LPS up-regulated/down-regulated gene signatures (*Lower Left*). (*I*) Clustered heatmap of the expression of CoV2 signature transcripts, displayed as FC over mean expression of mock for each condition and each batch. Each column represents one replicate. Column annotations indicate the infection condition. Row annotations indicate coregulated modules and their dominant composition, as well as transcripts belonging to ISG and LPS gene signatures.

Having observed a mixed inflammatory response to CoV2-infected epithelial cells in cocultured monocytes, we next asked whether it was specific to CoV2, by comparing monocyte responses to epithelial cells infected with either CoV2, IAV (another clinically significant respiratory pathogen of the orthomyxovirus family), or EBOV (a more distant nonrespiratory virus of the filovirus family, with a highly lethal hemorrhagic course also associated with strong inflammation; Fig. 2*A*). Epithelial infection levels were comparable between the three viruses (ranging from 30 to 80% in different IAV experiments, and ∼80% for EBOV; *SI Appendix*, Fig. S2 *A* and *B*).

IAV and EBOV both induced sizeable numbers of DEGs in cocultured monocytes (Fig. 2*B*), both viruses having roughly 50% stronger effects overall than CoV2. As for CoV2, the response to IAV infection in cocultured B cells and monocytes was very similar (*SI Appendix*, Fig. S2*C*). Direct comparison of monocyte transcriptional changes induced by CoV2 and IAV revealed that most down-regulated genes were shared between the two infections, while the up-regulated genes consisted of both shared and virus-specific modules (Fig. 2 *C*–*F*). The CoV2-specific component included several of the proinflammatory cytokines described above, especially *TNF* and *IL10*; *IL1B* was even down-regulated in IAV-infected cocultures (Fig. 2*C*). On the other hand, *IL6* and the granulocyte/monocyte stimulating factors *CSF2* and *CSF3* were equally induced by IAV and CoV2. A set of proinflammatory chemokines (*CCL3*, *CCL4*, and *CCL19*) were also up-regulated preferentially by CoV2 infection (Fig. 2*D*). The eosinophil chemotactic factor *CCL24* was among the genes most strongly down-regulated by both IAV and CoV2, suggesting that eosinophil recruitment is dampened in both infections. A substantial set of ISGs were induced at similar magnitudes by both viruses, but some ISGs also responded preferentially in the presence of CoV2 (Fig. 2*E*).

As discussed above, COVID-19 symptomatology includes several of the manifestations of sepsis, even in the absence of bacterial infection or obvious barrier breach. Furthermore, gene ontology analysis suggested that the CoV2 coculture signature harbored elements of innate activation through Toll-like receptor (TLR) 4 activation (Fig. 1*E*). To test this notion, PBMCs were incubated in parallel cultures with *Escherichia coli* lipopolysaccharide (LPS), a TLR2/4 ligand. The transcriptional signature of genes induced or repressed by LPS in monocytes superimposed strongly with CoV2-imparted changes (Fig. 2*F*). The LPS down-regulated gene set was largely common to CoV2 and IAV infections, while the up-regulated component of the LPS response was much more strongly influenced in CoV2- than in IAV-infected cocultures (median FoldChange [FC] = 1.37 vs. 0.95, χ^2^ *P* value [chisq *P*] ≤ 0.0001 vs. 0.24, respectively). Note that this intersection between the CoV2 coculture signature and TLR activation was not merely due to dead epithelial cells released in the culture: The transcriptional changes elicited in the monocytes by exposure to lysed HEK cells (killed by freeze–thawing) bore no relation to effects of CoV2- or IAV-infected cells (*SI Appendix*, Fig. S2*D*).

Calculating an index for responsive genes confirmed that, across monocytes from seven different HDs, the CoV2-down signature was equally elicited in CoV2 and IAV cocultures, but that the up signature was very specific to CoV2 (Fig. 2*G*). To exclude that this ineffectiveness of IAV-infected Caco-2 cells to induce the full ISG set was due to suboptimal infection, we profiled monocytes in cocultures with Caco-2 cells infected with a wide range of IAV multiplicity of infection (MOI). The CoV2-down signature was indeed most marked at an intermediate range (*SI Appendix*, Fig. S2*E*), but the CoV2-up signature could not be significantly induced at any MOI (in contrast, ISG induction was essentially linear to infection dose).

The comparison of monocytes in EBOV- and CoV2-infected cocultures largely reproduced the same themes (Fig. 2*H*): some degree of shared effects, particularly for down-regulated genes (quantitatively stronger for CoV2), and comparable induction of some antiviral ISGs, but a preponderance of virus-specific inductions. As in the IAV comparison, the key inflammatory cytokines and chemokines *TNF*, *IL1B*, *CCL3*, and *IL10* were uniquely induced by CoV2 (and even repressed by EBOV). In the EBOV cocultures, *IL6* transcripts were below the detection threshold. The LPS-induced signature showed branching into EBOV and CoV2 preferential induction, the latter being actually repressed in the EBOV cocultures (Fig. 2*H*). Overall, these results are recapitulated in the heatmap of Fig. 2*I* and Dataset S4, which also highlight the dichotomy between the two ISG-containing clusters (K2 and K4), only one of which was induced in all viral cocultures (K4), while the other is highly specific to CoV2 cocultures (K2; Fig. 2*I* and *SI Appendix*, Fig. S2*F*). Different ISGs are induced preferentially by type I IFN or IFNγ, and the ISGs of the shared cluster K4 predominantly corresponded to type I ISGs, while those of the CoV2-specific cluster K2 were enriched in IFNγ-responsive genes (*SI Appendix*, Fig. S2*G*).

In sum, coculture with CoV2-infected epithelial cells induces a complex response in monocytes, some of which is generic to all virus-infected cells, but most of which is quite specific to CoV2, in particular the proinflammatory moiety.

### Multiple CoV2 Proteins Can Partake in Triggering Cocultured Monocytes.

**Fig. 3.**
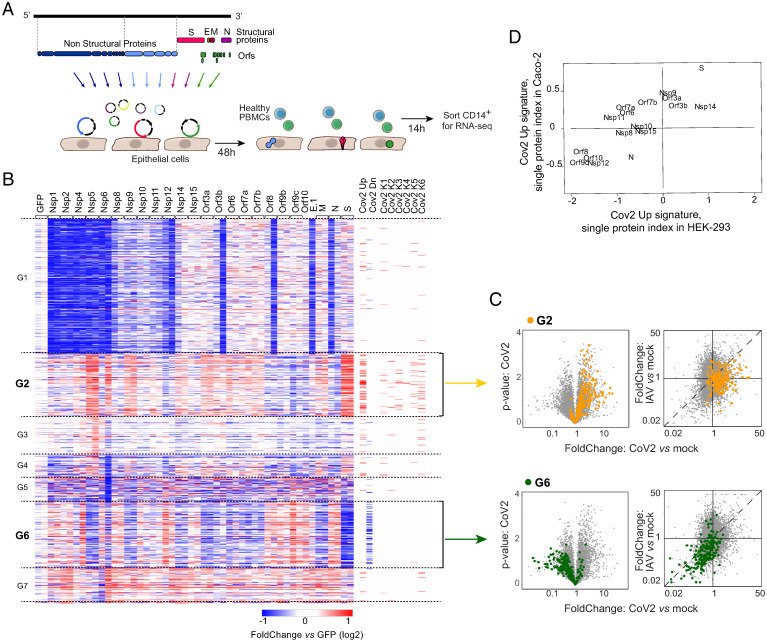
Part of the Cov2 signature can be recapitulated directly by the effects of individual CoV2 proteins. (*A*) Experimental approach. Twenty-seven CoV2 protein expression cassette plasmids or the GFP control protein expression cassette plasmid were transfected into Caco-2 or HEK cells, and 48 h or 24 h later, respectively, PBMCs from the same HD in each batch were added directly to the cultures. CD14^+^ monocytes were flow sorted 14 h later for population RNAseq. (*B*) Heatmap of the significantly differential expressed transcripts in monocytes cocultured with transfected Caco-2 (selection: overall variance in the dataset substantially exceeded interreplicate variance, and with significant difference from GFP-transfected controls in at least one coculture FC > 2 or < 0.5, *P* < 0.01). The columns represent the different transfected viral proteins (duplicates except for E [E.1]). Annotations at the right ribbon show the overlap between these genes and the Cov2 signature and its different clusters. (*C*) Overlay of clusters G2 (*Upper*, orange) and G6 (*Lower*, green) from heatmap in *B* onto viral coculture datasets: volcano plots of gene expression in monocytes cocultured with Cov2-infected Caco-2 compared to mock (*Left*; as in Fig. 1*A*) and FC–FC plots comparing the monocyte response to CoV2 relative to IAV (*Right*; as in Fig. 2 *C*–*F*). (*D*) Scatter plot of CoV2-up signature index in monocytes cultured with Caco-2 or HEK cells transfected with individual viral genes. Only conditions with at least two replicates passing the quality control are shown.

Given these specific effects of cells infected by CoV2, we then attempted to determine which viral proteins might be involved. As a screen, Caco-2 cells were transfected, in biological duplicates, with a panel of 27 plasmids encoding single viral proteins or GFP as a control (Fig. 3*A*). Forty-eight hours later, these transfectants were cocultured with HD PBMCs, and the monocytes were profiled by RNAseq after 14 h. Such transient transfections can be prone to technical artifacts from cell stress during transfection, plasmid DNA, or protein overexpression (42, 43). Indeed, the RNAseq data were noisy, with substantial variation between biological replicates. We thus selected a set of DEGs whose overall variance in the dataset substantially exceeded interreplicate variance, and with significant difference from GFP-transfected controls in at least one coculture. We then cross-referenced these genes to transcripts of the CoV2-induced signature. Although some genes with variable expression in cocultured monocytes showed no reproducible relation to effects in virus-infected cocultures, two groups of genes (G2 and G6 in Fig. 3*B*) had very strong overlap with the CoV2-up and CoV2-down gene sets. Several CoV2 proteins were able to up-regulate G2 and down-regulate G6 in the cocultured monocytes (most clearly, S, nsp5, nsp9, and nsp14), while others had a moderate repressive effect (N, nsp12, and orf9c). No notable level of cell death was induced by any of these plasmids, and the differential effects were reproducible in parallel experiments with independent plasmid preparations. Genes in G2 mostly corresponded to the proinflammatory and CoV2-specific clusters K5 and K6 defined in Fig. 2. On the other hand, the ISG component of the CoV2-up signature did not belong to this group but in a cluster with poor reproducibility and with little or no specific effects of S and nsp5 (*SI Appendix*, Fig. S3*A*). Thus, there was a disconnect between the proinflammatory and the ISG moieties of the CoV2-up signature: CoV2 proteins reproduced the inflammatory but not the ISG part.

Reciprocally, genes from G2 and G6 identified in the transfection cocultures proved almost entirely shifted in virus-infected cocultures (Fig. 3*C*). Here again, most up-regulated genes were not shared with IAV-infected cocultures, but the down-regulated signature was common to both (Fig. 3*C*).

For replication, we performed monocyte cocultures with transfection into a second epithelial cell line (HEK). Comparable patterns were observed (*SI Appendix*, Fig. S3*B*), with dominant effects of S, nsp9, and nsp14, but opposite effects of orf8, orf9, and orf10, which matched genes altered in both CoV2-infected and transfected Caco-2 cocultures (*SI Appendix*, Fig. S3*B*, top). The effects of individual CoV2 proteins showed generally concordant distribution after transfection in both cell lines (*SI Appendix* and Fig. 3*D*).

Thus, it was possible to replicate some of the monocyte response to CoV2-infected cells by expression of single viral proteins, confirming that the observed signatures were not merely confounders of infected cocultures, or induced by free viral RNA. Several proteins shared the same potential, implying that changes in monocytes were not due to viral proteins acting as specific triggers but, more likely, through changes that they induced in the infected epithelial cells. Active proteins settled into two groups, with diametrically opposite effects, which would presumably be balanced in the context of viral infection, but, overall, the virus best matched the S/nsp5/nsp14 group.

### Nontranscriptional Soluble Factors Account for the Coculture CoV2 Signature.

**Fig. 4.**
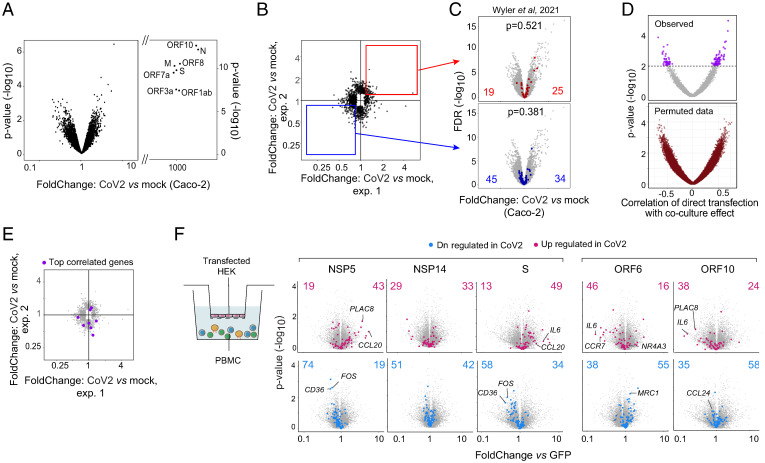
CoV2 and its proteins affect Caco2 cells nontranscriptionally. (*A*) Volcano plot of gene expression in CoV2-infected vs. mock-infected Caco-2 cells at 48 h post infection. CoV2 viral genes are labeled. (*B*) FC–FC plots comparing the Caco-2 response to CoV2 in two independent batches (points with nominal *P* value < 0.05 in either batch are shown). The red and blue squares highlight the consistent up- and down-regulated changes between the two batches (|log2FC| > 0.25 in same direction). (*C*) Overlay of reproducible genes from *B* onto volcano plot of CoV2- vs. mock-infected Caco-2 infected cells from Wyler et al. (44). *P* values of the overlaps were calculated using the χ^2^ test. (*D*) Per-gene correlation (Spearman) of transcriptional effect in Caco-2 cells transfected with individual viral genes with mean Cov2-up signature index in CD14 monocytes cocultured with transfected cells. Values obtained from data with randomized sample labels are shown on right. Nominal *P* values shown are based on permutation test after randomizing sample labels 100 times; purple highlighted genes: *P* < 0.01. (*E*) FC–FC plots comparing the Caco-2 response to CoV2 in two independents batches (as in *B*). The purple dots highlight the most correlated transcripts in the CoV2 protein transfected Caco-2 dataset (purple genes, *P* < 0.01, from *D*). (*F*) Overlap of the CoV2 signature in monocytes cocultured with transfected HEK in Transwells (HEK were cultured in the upper compartment, and PBMCs were added in the lower compartment). Volcano plots of gene expression in monocytes cocultured with transfected HEK (nsp5, nsp14, S, orf6, orf10) compared to monocytes cocultured with GFP-HEK in a Transwell setting. Part of the Cov2 signature significantly up- (red) or down-regulated (blue) in HEKs is highlighted and numbers are shown.

We then attempted to tackle the mechanistic pathway though which CoV2-infected or transduced Caco-2 cells elicit the CoV2 signature in healthy monocytes. We searched for candidate mediators by examining RNAseq profiles of CoV2-infected Caco-2 cells in our cultures. Few or no genes showed significant induction, except for viral proteins themselves (Fig. 4*A*). In an attempt to bring out minor effects, we aligned the results of two independent culture experiments (each in biological duplicate), and observed no enrichment in the concordant segment of the graph (Fig. 4*B*), suggesting that most of these low-significance signals were, indeed, noise. The few putatively reproducible changes in Fig. 4*B* did not show any bias in a previously published dataset of CoV2-infected Caco-2 cells (44) (Fig. 4*C*). Thus, in agreement with these authors, we conclude that CoV2 infection has surprisingly minor transcriptional effects in infected Caco-2 cells. We next generated RNAseq profiles from Caco-2 cells transfected with 27 individual viral genes, and searched for transcripts that would correlate, across all the transfectants, with the ability to induce the specific signature in cocultured monocytes. Very few transcripts showed significant correlation, with a distribution of correlation coefficient similar to that observed with random label permutation (Fig. 4*D*) and with no relationship between the most correlated transcripts and those putatively affected by CoV2 virus infection (Fig. 4*E*). We concluded that CoV2 and its proteins were inducing the activating potential in Caco-2 cells via nontranscriptional means.

To determine whether these CoV2-related effects were mediated by cell-to-cell contact or via soluble factor(s), we used a Transwell chamber to coculture monocytes and HEK cells transfected with a selected set of viral genes (Fig. 4*F*). Many of the CoV2-related effects were reproduced in this model, in particular, the comparable effects of nsp5, nsp14, and S, implying diffusible mediators for at least some of the CoV2-provoked effects. Thus, the epithelial response to infection by CoV2 virus, or by the enforced expression of its proteins, involves the generation of soluble mediators but not ones that are induced at the transcriptional level.

### The CoV2 Signature Carries to Monocytes of Severe COVID-19 Patients.

**Fig. 5.**
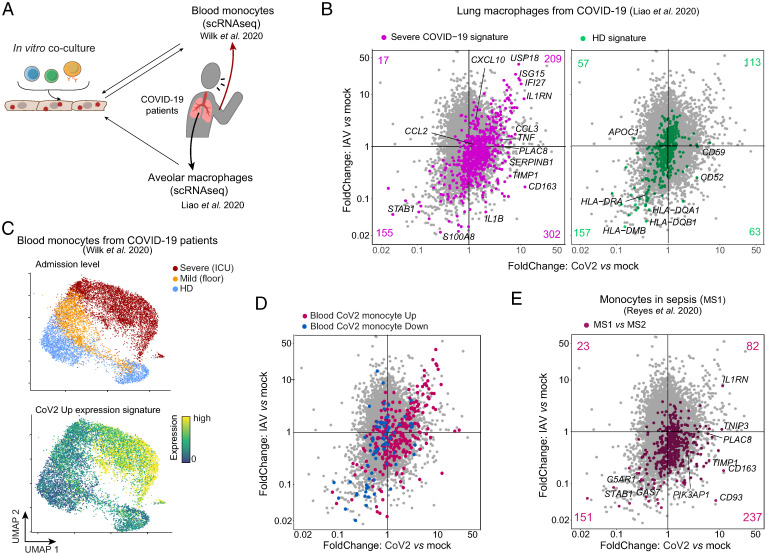
The CoV2 signature overlaps with myeloid signatures of severe COVID-19 patients. (*A*) Analysis scheme for comparison of the CoV2 monocyte coculture in vitro signature with gene expression datasets of published COVID-19 studies from systemic (blood monocytes) and local (lung macrophages) myeloid compartments. (*B*) FC–FC plots comparing the response of monocytes cocultured with CoV2-infected Caco-2 relative to monocytes cocultured with IAV-infected Caco-2. Highlighted gene signatures are from lung macrophages from severe COVID-19 patients (*Left*) or from HDs (*Right*) (19). (*C*) Two-dimensional UMAP representation of CD14+ monocyte single cell RNAseq (scRNAseq) extracted from GSE150728 (15). Each cell is color-coded by the patient’s severity at clinical admission (*Upper*) or by the expression of the CoV2-up signature genes (*Lower*). (*D* and *E*) FC–FC plots comparing the response of monocytes cocultured with CoV2-infected Caco-2 relative to monocyte cocultures with IAV-infected Caco-2, highlighted with gene signatures from (*D*) blood monocytes from severe COVID-19 patients (15) and (*E*) MS1 (sepsis state of monocytes) versus MS2 state (HLA-DR^hi^ monocytes) (45).

These observations raised the question of the biological and clinical relevance of these in vitro results to the in vivo setting. Do these results recapitulate perturbations described in COVID-19 patients? We extracted gene expression datasets of myeloid cells from published profiling studies of COVID-19 patients and looked for reciprocal enrichment of transcriptional effects (Fig. 5*A*; two studies were probed in detail, but shallower examination of other studies shows the conclusions to be generally applicable). First, in myeloid cells from the lungs of COVID-19 patients (19), whose contact with infected epithelia would most closely mimic our experimental configuration, gene expression signatures of alveolar macrophages from severe patients proved up-regulated in our CoV2-cocultured monocytes (Fig. 5*B*, *Left*; chisq *P* < 10^−4^); some aligned with the swath equally affected in IAV- and CoV2-infected cocultures (including ISGs like *IFI27* and *ISG15*), but the largest group belonged to the CoV2-specific quadrant (e.g., *IL1B*, *TNF*, *CCL3*, *CD163*, *TIMP1*, and *PLAC8*). On the other hand, genes overexpressed in macrophages from HD lung were unaffected or down-regulated in our datasets (Fig. 5*B*, *Right*; chisq *P* = 0.163). In blood monocytes (15), chosen to assess a systemic spread of the effect, the index computed from the coculture CoV2-up signature showed a clear correspondence with disease severity (Fig. 5*C*). In the other direction, the genes whose expression was up- or down-regulated in blood monocytes from these severe COVID-19 patients relative to unexposed controls showed a strong bias in our coculture datasets (Fig. 5*D*; chisq *P* < 0.006).

Given the described “sepsis without bacteria” clinical state of severe COVID-19 patients (3, 8) and the strong overlap between LPS-induced genes and our CoV2 coculture signature, we asked whether the CoV2 signature correlated with transcriptional alterations of the myeloid compartment in severe sepsis. Reyes et al. (45) reported an expansion of a specific monocyte state (MS1) in patients with severe bacterial sepsis, which was also up-regulated in monocytes from COVID-19 patients (21). Highlighting MS1 versus MS2 (classical MHC-II^high^ monocytes) signature genes in the coculture datasets revealed a significant enrichment in the CoV2-cocultured monocytes but a strong down-regulation from coculture with IAV-infected cells (Fig. 5*E*; chisq *P* < 10^−4^). Thus, the in vitro coculture CoV2 signature recapitulates the dysregulated myeloid state reported in severe COVID-19 patients, both at the local and systemic level, and overlaps with the bacterial sepsis monocyte profile.

### Monocytes from Children Have Muted Responses to CoV2.

**Fig. 6.**
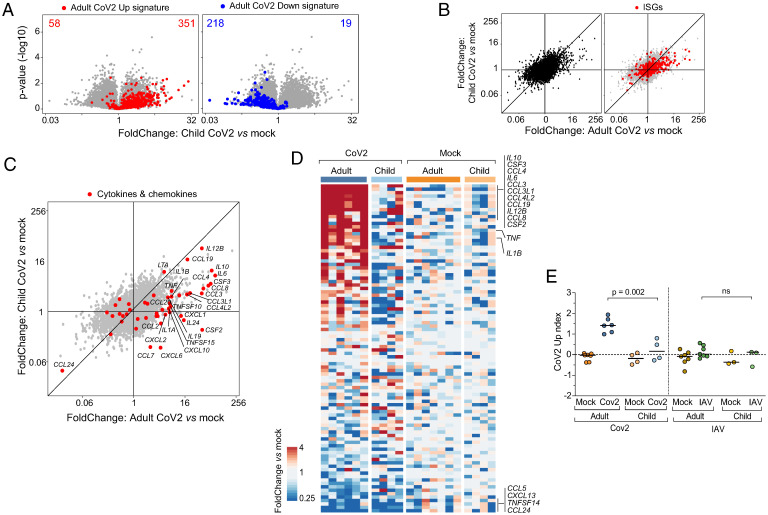
Monocytes from children have muted responses to CoV2. (*A*) Volcano plots displaying expression changes in children’s monocytes cocultured with CoV2- or mock-infected Caco-2 cells. The adult CoV2 up-regulated (red) and down-regulated (blue) gene signatures are highlighted. (*B* and *C*) FC–FC plots comparing the CoV2 response of children’s vs. adults’ cocultured monocytes, without highlight (*B*, *Left*) and highlighting the behavior of ISGs (*B*, *Right*) and cytokine and chemokine transcripts (*C*). (*D*) Heatmap of cytokine and chemokine induction in children’s and adults’ monocytes cocultured with CoV2-infected epithelial cells, displayed as FC over mean expression of mock for each condition and each batch. Each column represents one replicate. Column annotations indicate the infection condition. (*E*) CoV2 up-regulated gene index in children’s or adults’ monocytes cocultured with CoV2-, IAV-, or mock-infected epithelial cells. *P* values were calculated using the Mann–Whitney test. ns, not statistically significant.

Having observed a specific response to CoV2-infected cells in cocultured monocytes which corresponded to signatures in blood monocytes in severe COVID-19, we hypothesized that these effects might be related to age-dependent course of disease and the mostly benign evolution of COVID-19 in children. To test this notion, we cocultured PBMCs from healthy children (4 y to 14 y of age) with mock- or CoV2-infected Caco-2 cells, and compared monocyte transcriptional responses to those observed with adult monocytes (two independent BSL4 experiments). Analysis of the CoV2 up- and down-regulated signatures derived from adults showed that the monocyte response to CoV2 was qualitatively conserved in children (Fig. 6*A*). However, a direct comparison of adult and children monocyte responses showed a marked attenuation in children compared to adults, evidenced by the off-diagonal placement of most transcripts (Fig. 6*B*). This reduction applied to ISGs (Fig. 6*B*, *Right*) and to key cytokine and chemokine components of the monocyte response to CoV2 (Fig. 6 *C* and *D*). This shift was particularly marked for the major proinflammatory cytokines and chemokines induced by CoV2 in adult monocytes (*IL6*, *IL10*, *TNF*, *CCL3*, and *CCL4*), several of which were essentially flat in children’s monocytes relative to their controls (*SI Appendix*, Fig. S4). Calculating the CoV2 transcriptional index derived above confirmed that the CoV2-specific response in monocytes was significantly diminished in the monocytes of children, but that monocytes from adults and children were equally nonresponsive to IAV-infected cells (Fig. 6*E*). Thus, in this model of initial immune encounter with infected epithelial cells, monocytes from children react in a muted fashion compared to monocytes from adults, correlating with their lower susceptibility to severe COVID-19.

## Discussion

We developed an in vitro model of the initial encounter between immunocytes and CoV2-infected epithelial cells to investigate the cause of CoV2-induced immune changes. CoV2-infected epithelial cells directly stimulated a mixed antiviral and inflammatory response in monocytes, with components that were unique to CoV2 when compared to influenza virus and EBOV. Several CoV2 proteins could individually recapitulate parts of this proinflammatory response. A comparison of monocytes from adults and children demonstrated that children have quantitatively muted responses to CoV2-infected cells.

Returning to the question of why CoV2 induces such a unique pathological response in some patients, our results offer several suggestive insights. Supporting our in vitro results, this unique inflammatory component has also been observed in transcriptomic analyses from severe COVID-19 patients (19, 46), whereas it seemed absent in patients with severe IAV (47). This component was shared between B cells and monocytes. The strong proinflammatory character of monocytes cocultured with CoV2-infected cells, distinct from IAV and EBOV cocultures and reminiscent in many ways of a TLR-driven response, suggests that CoV2 may deviate the immune response at its earliest stages, at the expense of effective antiviral immunity. Such an idea would nod toward a mechanistic underpinning of the “sepsis without bacteria” clinical picture of COVID-19 that has been reported (3, 8). The induction of *IL6*, *TNF*, and *IL1B* is easy to consider in this context, but *IL10*, classically considered an antiinflammatory cytokine, is more puzzling. Like IL6, IL10 is strongly associated with COVID-19 severity (18, 48, 49), and some prior reports suggest that it may paradoxically enhance inflammation in such settings [IL10 enhances endotoxemia (50) and induces IFNγ in patients with Crohn’s disease (51)]. Later in the infection, this inappropriate initial polarization of the innate immune system may give rise to misfocused adaptive immune responses, such as the early germinal center exit of B cells and the poor T cell responses described in severe COVID-19 (18, 52, 53).

These results, and the dichotomy in ISG responses in CoV2 versus IAV/EBOV cocultures, also speak to the role of IFN and ISGs in COVID-19 pathogenesis, some aspects of which have been difficult to integrate. Type I IFNs are clearly important in controlling CoV2 infection, as deficiencies in IFN signaling, of either genetic or immunologic origin, are strong risk factors for severe COVID-19 (33, 34, 36, 37). Accordingly, many profiling studies have reported ISG up-regulation in PBMCs (for instance, refs. 15, 28, 47, and 54) or bronchoalveolar lavage (46) from severe COVID-19 patients. On the other hand, epithelial cells infected with CoV2 have been reported to produce low levels of type I IFNs (22, 55) relative to other viruses, and some in vivo studies have observed low type I IFNs in severe COVID-19 patients (22–24). In the cocultures, ISGs were induced along with the proinflammatory response, which is not surprising given that the response to CoV2-infected cells is highly reminiscent of the response to TLR ligands, and that IFN induction is a consequence of activation by many TLRs (via TRIF and IRF3). It was interesting that only half of the ISGs induced by CoV2-infected cells were shared with EBOV or IAV infection. This cluster, K4, included many key antiviral ISGs, and we propose that these correspond to true IFN-induced responses elicited by all viruses. On the other hand, the CoV2-specific ISGs of cluster K2 may be induced independently of Type-1 IFN, for example, by IFNγ or through other signaling pathways directly activated by infected epithelial cells. Thus, from the initial interaction between CoV2-infected epithelial cells and monocytes, the stage is set to counterbalance an IFN response that is essential for viral clearance by a proinflammatory diversion.

How do CoV2-infected cells stimulate this CoV2-specific response in monocytes, and what is the molecular mediator(s)? Our screen of individual CoV2 proteins and comparisons to LPS offer some clues: 1) The strong overlap with responses induced by LPS (a TLR2/4 ligand) implies that signaling pathways downstream of TLRs are being triggered in monocytes and B cells. 2) It is likely not the viral proteins themselves that activate these pathways, since several CoV2 proteins (as different as the ACE2-binding Spike protein and the viral protease nsp5) have superimposable abilities, this in Caco-2 as well as HEK cells, making it difficult to envisage this explanation. In addition, there is an interesting symmetry, where proteins like Orf8 or Orf9 actually repress S-induced genes (and induce S-repressed ones), suggesting two cellular states whose balance is perturbed by viral elements. 3) Our experimental setup should have avoided direct CoV2 infection of the monocytes themselves, and, indeed, few reads from the CoV2 genome were observed in the monocyte RNAseq (unlike IAV cocultures, where high viral reads suggested some degree of reinfection; Dataset S3). 4) Soluble mediators are at least partially involved, since cocultures with physical separation of the cells in Transwell reproduced these effects, but not simply by transcriptional induction of cytokine or chemokines, as evidenced by extensive profiling of the infected or transfected Caco-2 cells themselves. Integrating these threads, we suggest that CoV2 infection, and/or the expression of individual CoV2 proteins, causes the epithelial cells to display or release increased amounts of mediators that activate innate sensors in monocytes. Candidates include mediators such as HMGB1 (56), F-actin (57), or other cell-derived “damage-associated molecular patterns” (58). Our hypothesis that host products from infected cells trigger monocytes complements recent reports of a molecular interaction between CoV2 proteins and TLR2 or C-type lectins on myeloid cells (59, 60); some of us have also reported that the S protein from SARS-CoV-1, expressed in PBMCs via a herpes viral vector, can induce IL6 expression (40). This direct triggering by S may parallel the more general proinflammatory pathway induced by a variety of viral proteins, underlining the evolutionary importance of this response for highly pathogenic coronaviruses. Finally, Reyes et al. (21) have shown that IL6 alone can induce, in monocytes, transcriptional changes (the “MS1” program) with similarities to deviations observed in COVID-19 or sepsis patients, suggesting a causal role for IL6. This cannot be the case here (no *IL6* was detected in infected or transfected Caco-2 cells), but it may be that IL6 acts as a feed-forward loop, induced by CoV2-infected cells and then further amplifying the deviation.

Finally, what should we make of the muted response to CoV2-infected cells in monocytes from children, affecting both the ISG and proinflammatory components? The relative protection children enjoy from severe COVID-19 is one of the most unique aspects of CoV2 compared to other common respiratory viruses (11–13). Although this is only a two-point correlation, we speculate that the low responsiveness of their monocytes could be a key element of children’s relative protection. Mechanistically, immunocytes from children may be less responsive due to a relative naiveté vis-à-vis prior inflammatory exposures (a relative absence of “trained immunity”), or the difference may reflect the systemic proinflammatory tone that develops with aging (61, 62). It would be interesting to see whether differential responsiveness extends to B and other immunocytes as well, to distinguish an influence of monocyte maturity vs. shared influence of the overall milieu.

In conclusion, these results indicate that the dangerous inflammatory course followed by COVID-19 may be rooted in the very first immune interactions, with amplifying deviations that children are able to avoid. Modulating this inflammatory seed might prevent the subsequent exuberant and deleterious immune activation.

## Materials and Methods

### Viruses.

CoV2 stocks (isolate USA_WA1/2020), Influenza A PR8-GFP virus (A/Puerto Rico/8/1934(H1N1), and EBOV (isolate Mayinga) were grown in Vero E6 cells and purified by sucrose ultracentrifugation, and titers were determined. Work with EBOV and CoV2 was performed in the National Emerging Infectious Diseases Laboratories (NEIDL) BSL-4 facility. Caco-2 cells were seeded and infected 24 h later with CoV2 or EBOV at a nominal MOI of 10, with IAV at nominal MOI ranging from 0.1 to 10. After an adsorption period (2 h for CoV2 and EBOV, 1 h for IAV), the inoculum was removed and replaced with fresh media, and cells were incubated for 35 h prior to coculturing with PBMC.

### Transfections.

CoV2 expression plasmids were kindly provided by D. Gordon and N. Krogan (University of California, San Francisco, CA) (38). Two independent preparations of plasmids were used in independent transfection experiments. Twenty-four hours after seeding, epithelial cells were transfected using Lipofectamine 3000 (Thermo Fisher), washed after 8 h to 12 h, and grown for 24 h (HEK) or 48 h (Caco-2) before addition of PBMCs or lysis for RNAseq.

### Cocultures.

Deidentified PBMCs were analyzed, originating from 17 healthy adults (21 y to 65 y old) and 11 children (aged 4 y to 14 y), either before December 2019 or without recent COVID-19 symptoms plus negative PCR within 3 d prior. These experiments were performed under institutional review board (IRB) protocols IRB-P00021163, MBG2020P000955, and IRB15-0504. Thirty-five hours postinfection or 24 h to 48 h posttransfection, frozen PBMCs were thawed and washed, then added to washed epithelial cells for 14 h of coculture. For Transwell, HEK cells were transfected and replated onto Transwell inserts. Media from both chambers was replaced 24 h later, and PBMCs were added to the bottom chamber for 14 h of coculture. CoV2 and IAV cocultures were performed in three independent experiments (one pilot, one main experiment, and one replication experiment) with at least three biological replicates per condition (PBMCs from different donors). EBOV coculture was performed for one experiment with three PBMC sources. Transfectant cocultures were performed in two independent experiments, each including two biological replicates. Cocultured monocytes were purified by magnetic selection (if with virally infected cells) or by flow cytometry (if with transfected cells) prior to low-input RNAseq per ImmGen protocol (https://www.immgen.org). Viral reads were mapped to CoV2, EBOV, and IAV sequences from National Center for Biotechnology Information.

## Supplementary Material

Supplementary File

Supplementary File

Supplementary File

Supplementary File

Supplementary File

## Data Availability

The data reported in this paper have been deposited in the Gene Expression Omnibus database under accession GSE186650. Further details are available in *SI Appendix*, *SI Materials and Methods*.

## Change History

January 22, 2022: The byline has been updated.
