# Supplementary material for: A virus-specific monocyte inflammatory phenotype is induced by SARS-CoV-2 at the immune–epithelial interface
Source: Proc Natl Acad Sci U S A. 2021 Dec 28;119(1):e2116853118. doi: 10.1073/pnas.2116853118 (PMC8740714; doi:10.1073/pnas.2116853118)
Supplement: Supplementary File [file pnas.2116853118.sapp.pdf]

PNAS
www.pnas.org

Supplementary Information for

A virus-specific monocyte inflammatory phenotype is induced by SARS-CoV2 at the immune-epithelial interface

Juliette Leon[1,2*], *Daniel A. Michelson*[1*], *Judith Olejnik*[3,4*], *Kaitavjeet Chowdhary*[1*], *Hyung Suk Oh*[5*], *Adam J. Hume*[3,4], *Silvia Galván-Peña*[1], *Yangyang Zhu*[1], *Felicia Chen*[1], *Brinda Vijaykumar*[1], *Liang Yang*[1], *Elena Crestani*[6], *Lael M. Yonker*[7], *David M. Knipe*[5], *Elke Mühlberger*[3,4] and *Christophe Benoist*[1,+]

[1] Department of Immunology, Blavatnik Institute, Harvard Medical School, Boston, MA, USA
[2] INSERM UMR 1163, University of Paris, Institut Imagine, Paris, France
[3] Department of Microbiology, Boston University School of Medicine, Boston, MA, USA
[4] National Emerging Infectious Diseases Laboratories, Boston University, Boston, MA, USA
[5] Department of Microbiology, Blavatnik Institute, Harvard Medical School, Boston, MA, USA
[6] Division of Immunology, Boston Children's Hospital and Department of Pediatrics, Harvard Medical School, Boston, MA, USA
[7] Department of Pediatrics, Massachusetts General Hospital, Boston, MA, USA

* Equal contribution

This PDF file includes:

Supplementary text
Figures S1 to S4
SI References
Dataset legengs

SI MATERIALS AND METHODS

Peripheral blood mononuclear cells samples

PMBC samples from 17 healthy adults (21 to 65 years-old) and 11 children (4 to 14) were all collected either before December 2019, or with no recent symptoms consistent with COVID-19 and a negative PCR test within 3 days prior to collection. Some samples were purchased frozen from AllCells (Alameda,CA,USA), or were residual materials from prior studies at Boston Children's Hospital (EC), Mass General Hospital (LY) or Harvard Medical School (CB). These experiments were performed under IRB protocols IRB-P00021163, MBG2020P000955 and IRB15-0504.

Cells and Cell culture

HEK (ATCC CRL-1573) and Caco-2 cells (ATCC HTB-37) were cultured in Dulbecco's modified Eagle's medium (DMEM, Gibco) with 10% FBS, penicillin (50 U/ml), streptomycin (50 mg/ml), and 1% MEM non-essential amino acids in a humidified incubator at 37°C with 5% CO2. Cells were passaged using 0.05% Trypsin-EDTA solution. Cultures were verified to be free of mycoplasma contamination using MycoAlert Mycoplasma Detection Kit (Lonza).

CoV2 and EBOV propagation and titration

SARS-CoV2 stocks (isolate USA_WA1/2020, kindly provided by CDC's Principal Investigator Natalie Thornburg and the World Reference Center for Emerging Viruses and Arboviruses (WRCEVA)) and EBOV (isolate Mayinga, kindly provided by Heinz Feldmann, NIH NIAID Rocky Mountain laboratories) were grown in Vero E6 cells (ATCC CRL-1586) and

cultured in DMEM supplemented with 2% FBS, penicillin (50 U/ml), and streptomycin (50 mg/ml). To remove confounding cytokines and other factors, viral stocks were purified by ultracentrifugation through a 20% sucrose cushion at 80,000 x g for 2 h at 4°C (1). Viral titers were determined in Vero E6 cells by tissue culture infectious dose 50 ($TCID_{50}$) assay and calculated using the Spearman-Kärber algorithm.

All work with EBOV and CoV2 was performed in the biosafety level 4 (BSL-4) facility of the National Emerging Infectious Diseases Laboratories at Boston University, Boston, MA following approved SOPs and inactivation procedures.

Influenza A virus propagation and titration

Influenza A PR8-GFP virus (A/Puerto Rico/8/1934(H1N1), hereafter IAV) and Madin-Darby canine kidney (MDCK) cells were provided by D. Lingwood (Ragon Institute). IAV stock was made using MDCK cells and titer was determined by plaque assay as described previously (2).

Viral infection of Caco-2 cells for co-culture

One day prior to infection, Caco-2 cells were seeded at a density of 10^5 cells per well of a 24-well tissue culture plate or 25.10^3 cells per well of a 96-well tissue-culture plate. Twenty-four hours later, cells were infected with CoV2 or EBOV at a nominal MOI of 10, with IAV at nominal MOI ranging from 0.1-10. After an adsorption period (2h for CoV2 and EBOV, 1h for IAV), the inoculum was removed, replaced with fresh media (2% FBS supplemented DMEM), and cells were incubated at 37˚C for 35 h prior to coculturing with PBMC.

Plasmids and transfections

Expression plasmids were kindly provided by D. Gordon and N. Krogan (UCSF, San Francisco): 27 plasmids encoding CoV2 single viral proteins and GFP as a control (3). Most proteins were cloned in a pLVX-Puro backbone under an EF1a promoter; Spike was cloned in a pTwist backbone under an EF1a promoter. They were transformed into One Shot™ Stbl3™ competent *E. Coli* (Thermo Fisher) and were amplified using Zippy plasmid miniprep kits (Zymo Research). Two independent preparations of plasmids were made and used in independent transfection experiments. Epithelial cells, in suspension (Caco-2) or seeded 24 h prior (HEK), at a concentration of 25.10^{3} cells/well, were transfected with 100 ng of each plasmid using Lipofectamine™ 3000 reagent (Thermo Fisher) in Opti-MEM™ medium (Gibco) and washed 8 – 12 h later. They were then grown for 24 h (HEK) or 48 h (Caco-2) in 96-well flat-bottom tissue culture plates before addition of PBMCs or direct lysis for RNAseq

Coculture of PBMCs with infected or transfected epithelial cells

The same experimental design was used for viral-co-cultures and transfectant-co-cultures. 35 h post-infection or 24-48 h post-transfection, adherent epithelial cells were washed gently twice with standard culture media (DMEM, 10% FBS, penicillin (50 U/ml), streptomycin (50 mg/ml), and 1% MEM non-essential amino acids) to remove cell debris. Frozen PBMCs were thawed in a 37°C water bath for 90-120 sec then added dropwise to 9 mL of pre-warmed culture medium and centrifuged at 300 x g for 7 min. After removing supernatant, the PBMC pellet was resuspended in pre-warmed culture medium at a concentration of $1.5.10^{6}$ cells/ml. This PBMC suspension was slowly added to the epithelial cells at a final concentration of $7.5.10^{5}$ PBMCs/well (24-well plate,

infections) or 2.10^5 PBMCs/well (96-well plate, transfections). PBMCs and epithelial cells were then co-cultured for 14 h in a 37°C incubator (5% CO2).

For Transwell transfectant experiments, HEK cells were transfected in standard flat-bottom plates, dissociated into single cell suspension, and re-plated at 30,000 cells per well onto 24-well Transwell polyester 0.4 µm pore membrane inserts (Corning) with media filling both top and bottom chambers. Twenty-four hours after seeding, media from both chambers was replaced and 2.10^5 freshly thawed PBMCs were added to the bottom chamber for 14 h co-culture.

CoV2 and IAV co-cultures were performed in three independent experiments (one pilot, one main experiment and one replication experiment) with at least 3 biological replicates per condition. EBOV co-culture was performed for one experiment with 3 independent replicates. Transfectant co-culture were performed in two independent experiments for each cell line (each experiment used a different preparation of plasmid DNA), and each experiment included 2 biological replicates for each transfected protein.

Cell treatments

To determine the response to LPS, PBMCs were cultured for 14 h, in parallel to HEK co-cultures, with or without 1 ng/mL of LPS (LPS from E. coli O55:B5, Sigma, Cat# L2880) for 14 h. To assess the effects of co-culture with lysed cells, a freshly passaged single cell suspension of HEK cells at $2.5.10^5$ cells/ml was frozen on dry ice, then thawed rapidly at 37°C. The freeze-

thawed suspension was centrifuged at 500 x g to eliminate debris and added at a 10-fold dilution in PBMC cultures for 14 h.

Magnetic isolation of PBMCs in viral co-cultures

Due to lack of flow cytometry sorting in the BSL-4 containment laboratory, different PBMC subpopulations were isolated by magnetic sorting. Briefly, after 14 h of co-culture, supernatants containing PBMCs were harvested and washed, and dead cells were removed using the EasySep™ Dead Cell Removal (Annexin V) Kit (StemCell, #17899). The cell populations of interest, monocytes ($CD14^{+}CD16^{-}$) or B cells ($CD19^{+}$), were isolated by negative magnetic selection following manufacturer's instructions, using EasySep™ Human Monocyte Isolation Kit (StemCell, #19359) and EasySep™ Human B Cell Isolation Kit (StemCell, #17954). The isolated samples were resuspended in lysis buffer (TCL Buffer (QIAGEN) supplemented with 1% 2-mercaptoethanol) at a concentration of 500-1,500 cells per 5 µl, and frozen in a DNA Lo-Bind tube (Eppendorf, #022431021) at -80°C. For EBOV and CoV2 co-cultures, samples were heat-treated for 45 min at 60°C, removed from the BSL-4 laboratory and stored at -80°C.

In parallel, Caco-2 cells seeded in a 96-well plate were infected with one of the three viruses and cultured alone. At 48 h post infection (similar harvesting timepoint as for co-cultures), the cells were washed and directly lysed in 125 µL of TCL/βME lysis buffer, and heat-treated as above.

Flow sorting of PBMCs in transfectant co-cultures

PBMCs were harvested after 14 h co-culture, washed twice and stained using CD14 Pacific Blue (clone M5E2, BioLegend cat# 301815, 3:100); CD19 PerCP-Cy5.5 (clone H1B19,

BioLegend #302230, 3:100); CD3 Alexa Fluor 700 (clone OKT3, BioLegend, #317340, 2:100); CD45 APC-H7 (clone 2D1, BD, #560178, 2:100). Monocytes and B cells were immediately sorted as DAPI– $CD45^{+}CD3^{-}CD19^{-}CD14^{+}$ and DAPI-CD45+CD3-CD19+ respectively on a Moflo Astrios sorter (Beckman Coulter). 1,000 cells were sorted directly into 5 μl TCL/βME lysis buffer.

Assessment of infection rate in epithelial cells

Flow cytometry: After 14 h of co-culture and the removal of the supernatant containing PBMCs, epithelial cells were harvested after trypsinisation and stained in PBS with Zombie UV viability dye (BioLegend, #423107, 1:500) for 10 min. Then, cells were fixed and inactivated in 10% formalin (Fisher Scientific) for a minimum of 6 h at 4°C and removed from the BSL-4 laboratory. Fixed cells were washed in staining buffer (PBS + 2% FBS + 1mM EDTA) and permeabilized in 0.1% saponin buffer for 10 min at room temperature (RT). After blocking the non-specific binding sites by incubation in blocking buffer (PBS + 10% of donkey serum) for 1 h, cells were stained (2 h, RT) with either: anti-GFP AlexaFluor488 (clone FM264G, BioLegend, #338008, 1:200), rabbit anti-SARS-CoV nucleocapsid (N) protein (Rockland, #200-401-A50, 1:500, cross-reacts with the CoV2 nucleocapsid protein) or goat anti-EBOV VP35 protein (custom-made by Antagene, 1:200). For EBOV and CoV2, cells were stained 30 min with the secondary antibodies anti-rabbit and anti-goat AlexaFluor488, respectively (both 1:500, Jackson ImmunoResearch). Flow cytometry was performed on a FACSymphony™ flow cytometer (BD Biosciences) and analysed using FlowJo 10 software.

Immunohistochemistry: For CoV2 and EBOV, Caco-2 cells seeded in 96-well plates were infected as described above. One day post-infection, cells were fixed and inactivated in 10% formalin **(**Fisher Scientific**)** for a minimum of 6 h at 4°C and removed from the BSL-4 laboratory.

The cells were permeabilized with acetone-methanol solution (1:1, vol:vol) for 5 min at -20°C, incubated in 0.1 M glycine for 10 min at RT and incubated in blocking reagent (2% bovine serum albumin, 0.2% Tween 20, 3% glycerin, and 0.05% sodium azide in PBS) for 20 min at RT. After each step, the cells were washed 3 times in PBS. The cells were incubated overnight at 4°C with a rabbit antibody recognizing CoV2 N protein (Rockland, #200-401-A50; 1:1000) and a goat-anti-EBOV VP35 antibody (custom-made by Antagene, 1:200) in Cov2 and EBOV infection respectively. The cells were washed 4 times in PBS and incubated with goat anti-rabbit antibody conjugated with AlexaFluor488 or donkey-anti-goat antibody conjugated with AlexaFluor594 for 1 h at RT (Invitrogen; 1:200). 4',6-diamidino-2-phenylindole (DAPI; Sigma-Aldrich) was used at 200 ng/mL for nuclei staining. Images were acquired using a Nikon Eclipse Ti2 microscope with Photometrics Prime BSI camera and NIS Elements AR software.

For IAV, PR8-GFP-infected and PBMC-added Caco-2 cells were directly imaged live at 48 h post-infection using Nikon TE200 and SPOT imaging software (v5.2).

Population Low-input RNA-seq

Low-input RNA-seq was performed following the standard ImmGen low-input protocol (www.immgen.org), from the 5µl of collected lysis buffer. For the viral co-cultures, magnetically isolated samples were centrifuged at maximum speed (17,000 x g) for 3 min at 4°C before loading, in order to pellet the cellular debris and magnetic particles, the cleaned RNA lysate remaining in the supernatant. Smart-seq2 libraries were prepared as described previously (4) with slight modifications. Briefly, total RNA was captured and purified on RNAClean XP beads (Beckman Coulter). Polyadenylated mRNA was then selected using an anchored oligo(dT) primer (50 – AAGCAGTGGTATCAACGCAGAGTACT30VN-30) and converted to cDNA via reverse

transcription. First strand cDNA was subjected to limited PCR amplification followed by Tn5 transposon-based fragmentation using the Nextera XT DNA Library Preparation Kit (Illumina). Samples were then PCR amplified for 12 cycles using barcoded primers such that each sample carries a specific combination of eight base Illumina P5 and P7 barcodes for subsequent pooling and sequencing. Paired-end sequencing was performed on an Illumina NextSeq 500 using 2 x 38bp reads with no further trimming.

RNA-seq data processing and QC

Reads were aligned to the human genome (GENCODE GRCh38 primary assembly and gene annotations v27) with STAR 2.5.4a (https://github.com/alexdobin/STAR/releases). The ribosomal RNA gene annotations were removed from the GTF (General Transfer Format) file. The gene-level quantification was calculated by featureCounts (http://subread.sourceforge.net/). Raw read counts tables were normalized by median of ratios method with DESeq2 package from Bioconductor (https://bioconductor.org/packages/release/bioc/html/DESeq2.html) (5) and then converted to GCT and CLS formats. To map the viral reads, the SARS-CoV2, EBOV and PR8 IAV genome sequences and annotations were obtained from NCBI, with accession numbers: GCF_009858895.2, GCF_000848505.1, GCF_000865725.1. Reads were aligned by STAR 2.5.4a, with, for SARS-CoV2, specific parameters suggested in Kim *et al*.(6).

Quality control: Samples with less than 1 million uniquely mapped reads were automatically excluded from normalization to mitigate the effect of poor-quality samples on normalized counts. Samples having fewer than 8,000 genes with over ten reads were also removed from the data. We screened for contamination by using known cell type specific transcripts (per ImmGen ULI RNAseq and microarray data). Finally, the RNA integrity for all samples was

measured by median Transcript Integrity Number (TIN) across human housekeeping genes with RSeQC software (http://rseqc.sourceforge.net/#tin-py). For co-cultures with virally infected cells, samples with TIN <30 were removed from the BSL4 datasets prior to downstream analysis (the usual threshold, TIN <45, was used elsewhere). To eliminate unreliable datasets in the transfection analysis, samples with inter-replicate correlation <0.92 were omitted. To avoid quantitatively unreliable values from low expression, only genes with a minimum read count of 20 TPM in at least two samples were retained for analysis.

Differential gene expression and consensus CoV2 signature: We used an uncorrected t-test (comparing log-transformed expression values in monocytes of CoV2- vs mock-infected co-cultures) to assess differential gene expression between the different groups from the normalized read counts dataset. Genes with a FC >2 or <0.5 and nominal p-value < 0.01 were selected for some analyses (FigS1, FigS2B). A reproducible CoV2 signature set was computed by integrating the results from the first 2 co-cultures experiments (adult PBMCs with CoV2- or mock-infected Caco-2 cells). We first flagged genes which passed CoV2/Mock FC>2 and t.test -log10 (p.value) >1.5 in each experiment (and FC<0.5 for the down signatures). The intersect identified 123 transcripts matching in both experiments, but it was clear that this stringent selection, associated with the low numbers of genes reliably detected in Experiment1, were setting aside too many truly affected transcripts. We thus supplemented this list by including transcripts with CoV2/Mock FC>1.8 and correlated with the selected geneset (as mean of normalized expression across all cells) at Pearson r>0.85, yielding a cov2 signature of 429 up- and 246 downregulated transcripts, which was used as a standard in later experiments. The signature geneset was replicated to 87% in the third independent experiment. FC values (vs the mean of mock-infected control datasets) for these signature genes were imported into Morpheus (Broad Institute,

https://software.broadinstitute.org/morpheus), in which k-means clustering was performed with empirically chosen k (clustering used in Fig.2I).

Computation of module indices: the CoV2 index was calculated for each donor by averaging the log2 normalized expression (versus mean of all mock) of all genes belonging to the CoV2 signature (subtracting the average of the up signature to the down signature)

Analysis of the transfectants co-culture: Datasets from co-cultured monocytes or B cells were pre-processed as above. For Caco-2 transfectants (Fig. 3), QC-passing datasets were obtained for 27 single-protein datasets (+ GFP transfected controls), all in duplicates. We first selected transcripts with relevant variation by comparing (i) the vector of inter-replicate coefficients of variation (CV), a reliable indicator of experimental noise (computed as the mean of all inter-replicate CVs, this for every gene) with (ii) the overall CV within the dataset (averaged from CV computed from 1,000 randomly picked dataset pairs, excluding replicates). Transcripts were selected if overallCV-replicCV>0.2 OR (replicCV<0.4 & ((overallCV>(replicCV*2)), and their FC values (vs the mean of co-cultures with GFP-transfected cells) imported into Morpheus for heatmap representation and clustering (Fig. 3B, split into 7 empirically determined k-means clusters). The same procedure was followed for HEK transfectants (SI Appendix, Fig. S3B).

Correlation analysis of transfected Caco-2 cells with corresponding co-culture effects: To search for transcripts in transfected Caco-2 cells associated with induction of the CoV2-Up signature in co-cultured monocytes, we first constructed a coculture effect vector by computing the mean log2 fold change vs GFP controls of genes belonging to the CoV2-Up signature in monocytes co-cultured with Caco-2 cells transfected with individual viral genes. Using matched transfectant samples, we computed a per-gene correlation (Spearman) between this vector and the log2 fold change in Caco-2 cells transfected with individual viral genes vs GFP controls. To assess

the statistical significance of the resulting correlation coefficients, we repeated this procedure after permuting sample labels 100 times. As the correlation coefficients from the permutation were approximately normally distributed, p values were calculated using a two-tailed one-sample z-test using the mean and standard deviation of the coefficients from permuted data for each gene.

Geneset enrichment analysis: Enrichment of CoV2.up and CoV2.dn signature in Gene Ontology (GO) biological processes pathways and Reactome pathways was computed using Fisher's exact test with BH-FDR correction, through the g:Profiler interface. Pathways with a false discovery rate (FDR) <1% were selected for further analyses and recorded in Dataset S2. In order to overcome the inherent redundancy of GO pathways, significant pathways were then interpreted and visualized as an enrichment network using Cytoscape (v3.8.2) (7) and its *EnrichmentMap* and *AutoAnnotate* modules. Briefly, Cytoscape allows collapsing of redundant significant pathways into a single biological theme using the Jaccard Overlap combined index (cutoff=0.5), thus simplifying the interpretation. After filtering noise, the network represents overlaps among the most enriched pathways (FDR 0.5%), in which similar pathways are automatically group into main biological themes. Regarding the type I IFN and IFN gamma signatures, data were downloaded from the Gene Expression Omnibus (GEO) (https://www.ncbi.nlm.nih.gov/geo/) from two human published datasets GSE142672 and GSE46599 (8). To reduce noise, genes with a CV between biological replicates <0.7 and an expression level >20 in either comparison groups were selected. Upregulated transcripts were defined, at an arbitrary threshold, as having a FC >1.5 and a t-test p-value <0.05.

Overlap with COVID-19 patient profiling datasets: Signatures from *in vivo* myeloid population were extracted from published sources. The alveolar macrophage signature from severe COVID-19 patients was extracted from Liao et al. (9), by merging the signature of their two

predominant populations found in severe patients: FCN1^{hi} (group1) and FCN1^{lo}SPP1^{+} (group2). Signature of the MS1 state in sepsis was directly downloaded from the supplementary tables of Reyes et al (10). PBMC datasets from (11) were retrieved from the COVID19 cell atlas at https://www.covid19cellatlas.org/index.patient.html as an R dataset object which included the cellXgene matrix, and were used with the cell annotation provided by Wilk et al. The CD14^{+} monocyte cluster was extracted; indices for the CoV2-up signature genes were computed by averaging the pre-computed CoV2-up genes together per cell, and color-coded on the monocyte UMAP space for Fig.4C.

Statistical analysis

Unless specified otherwise, the data are presented as mean ± SD and tests of associations for different variables between infected/transfected and mock/GFP were computed using the nonparametric Mann Whitney test. Significance of signature overlaps into our dataset was assessed by Chi square test when computing one signature at a time (e.g. assessing one signature in an independent volcano plot), or by a Fisher's exact test with BH-FDR correction when using large curated GO signatures databases. Analyses and plots were done using S-Plus (v8.2.0), RStudio (v.1.2.5019) and GraphPad Prism (v.8.4.3), heatmaps generated with Morpheus (https://software.broadinstitute.org/morpheus).

SI REFERENCES

1. J. Olejnik, et al., Ebolaviruses associated with differential pathogenicity induce distinct host responses in human macrophages. *J. Virol.* **91,** (2017).

2. K. J. Szretter, A. L. Balish, J. M. Katz, Influenza: propagation, quantification, and storage. *Curr. Protoc. Microbiol.* **Chapter 15,** Unit (2006).

3. D. E. Gordon, et al., Comparative host-coronavirus protein interaction networks reveal pan-viral disease mechanisms. *Science* **370,** (2020).

4. S. Picelli, et al., Full-length RNA-seq from single cells using Smart-seq2. *Nat Protoc.* **9,** 171-181 (2014).

5. M. I. Love, W. Huber, S. Anders, Moderated estimation of fold change and dispersion for RNA-seq data with DESeq2. *Genome Biol.* **15,** 550 (2014).

6. D. Kim, et al., The architecture of SARS-CoV-2 transcriptome. *Cell* **181,** 914-921 (2020).

7. P. Shannon, et al., Cytoscape: a software environment for integrated models of biomolecular interaction networks. *Genome Res.* **13,** 2498-2504 (2003).

8. C. Goujon, et al., Human MX2 is an interferon-induced post-entry inhibitor of HIV-1 infection. *Nature* **502,** 559-562 (2013).

9. M. Liao, et al., Single-cell landscape of bronchoalveolar immune cells in patients with COVID-19. *Nat. Med* **26,** 842-844 (2020).

10. M. Reyes, et al., An immune-cell signature of bacterial sepsis. *Nat. Med* **26,** 333-340 (2020).

11. A. J. Wilk, et al., A single-cell atlas of the peripheral immune response in patients with severe COVID-19. *Nat. Med.* **26,** 1070-1076 (2020).

SI FIGURE LEGENDS

Fig S1.

(A) Representative flow cytometry plots showing a sample of the final isolated fractions of magnetically-purified monocytes (top) and B cells (bottom). Data from one set-up co-culture experiment.

(**B**) Volcano plots displaying gene expression changes in CoV2-infected Caco-2 cells versus mock Caco-2 cells, 48 h post-infection. On the left panel are highlighted the differentially expressed genes ($p<0.01$, FC>2 or <0.5). On the right panel are highlighted the CoV2 upregulated (red) and downregulated (blue) gene signatures. P-values were calculated using the Chi square test.

(**C**) Volcano plot of gene expression in B cells co-cultured with CoV2 infected Caco-2 compared to monocytes co-cultured with uninfected Caco-2 (mock). Differentially expressed genes are highlighted ($p<0.01$, FC>2 or <0.5).

Fig S2.

(**A-B**) Infection rate of IAV and EBOV in Caco-2 cells assessed: **(A)** by flow cytometry at the time of harvesting PBMCs (~48 h post-infection, one sample of the third independent experiment for IAV) **(B)** by immunofluorescence microscopy at 1 day post-infection for EBOV (left panel, 10X magnification) or at the time of harvesting PBMCs for IAV (~48 h post-infection in the setting of co-culture PBMCs added to infected epithelial cells, one sample of the second independent experiment, right panel). Infected cells are marked in green (GFP-PR8) for IAV and in red (anti-VP35 + anti-goat-AF594) for EBOV. Nuclei are marked with DAPI (blue, EBOV) or cellular membranes visible in brightfield (IAV).

(**C**) FoldChange-FoldChange plot comparing the response of B cells versus monocytes in the context of IAV infection in Caco-2.

(**D**) Overlay of transcriptional signature derived from monocytes co-cultured with killed epithelial cells onto viral co-culture datasets. Left panel shows the gene expression changes induced in monocytes co-cultured with killed epithelial cells (freeze-thawed HEK, see methods) highlighting the differentially expressed genes ($p<0.01$, FC>2 or <0.5). Right panel shows these genes into the FoldChange-FoldChange plot CoV2 versus IAV in monocytes.

(**E**) Changes in gene expression as a function of MOI of IAV infection in monocytes co-cultured with IAV infected Caco-2. Each gene is a dot. Genes belonging to the CoV2 down signature (left panel), the CoV2 up signature excluding the 2 ISG clusters (middle panel) or the two ISGs clusters (right panel) are shown.

(**F**) Dichotomy in the IFN response between IAV and CoV2 highlighted in a FoldChange-FoldChange plot comparing the response in monocytes co-cultured with IAV (y-axis) relative to CoV2 (x-axis) infected Caco-2**.** K2 (orange) and K4 (yellow) are highlighted.

(**G**) Same plots than in (F), highlighting: upregulated transcripts from GSE46599 (type I IFN-treated THP-1 cells for 24h), left panel, and GSE142672 (IFN gamma-treated blood monocytes for 24h), right panel.

Fig S3.

(**A**) Heatmap of ISG transcript expression values in the Caco-2 transfectant dataset.

(**B**) Heatmap of the significantly differential expressed transcripts in monocytes co-cultured with transfected HEK (similar selection as for Caco-2: overall variance in the dataset substantially exceeded inter-replicate variance, and with significant difference from GFP-transfected controls

in at least one co-culture FC>2 or <0.5, p<0.01). The columns represent the different conditions, where only proteins for which both replicates passed the QC are shown. Annotations at the right ribbon show the overlap between these genes and part of the CoV2 signature that was up/down regulated in Caco-2.

Fig S4.

Representation of the average expression of a selected set of cytokines across all CoV2-co-cultured monocyte replicates (mean and SEM), child (upper panel) or adult (lower panel). Each individual replicate is a dot. Score computed independently for each cytokine, where 0 corresponds to the average expression of this cytokine in the respective mock condition and 1 its average expression in CoV2-co-cultured adult monocytes (red line). The annotated p-values were computed from the values in children vs adult samples for each cytokine, using the Mann–Whitney test.

Figure S1

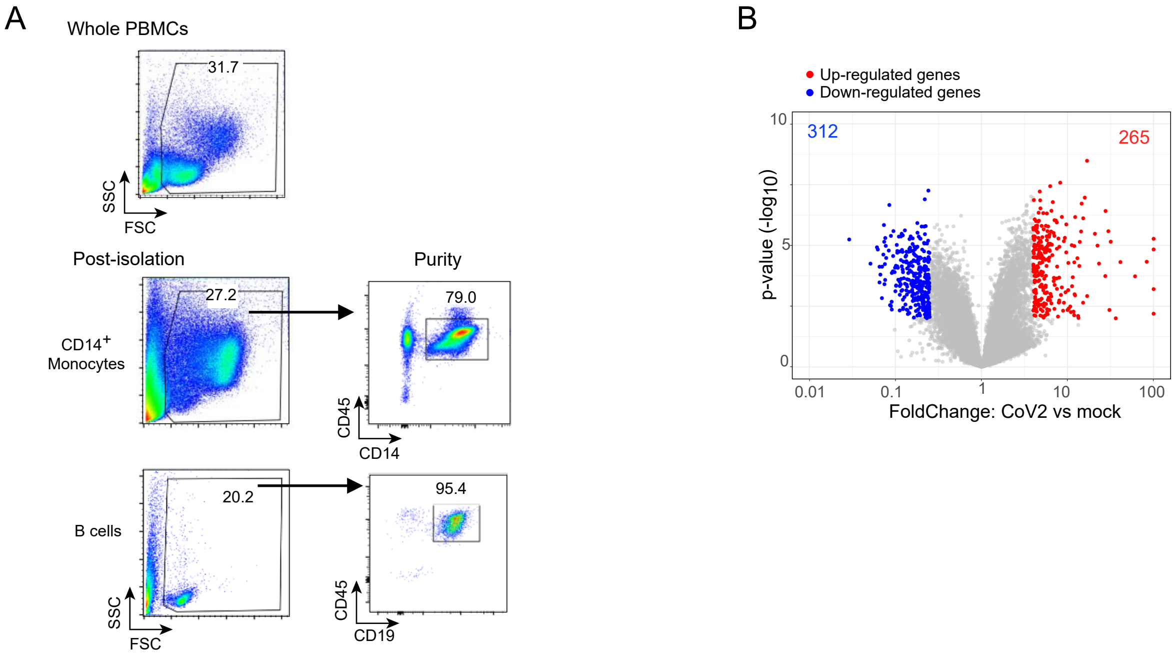
A
Whole PBMCs
31.7
SSC
FSC
Post-isolation
Purity
27.2
79.0
CD14+
Monocytes
CD45
CD14
20.2
95.4
B cells
SSC
FSC
CD45
CD19
B
Up-regulated genes
Down-regulated genes
312
265
p-value (-log10)
10
5
0
0.01
0.1
1
10
100
FoldChange: CoV2 vs mock

Figure S2

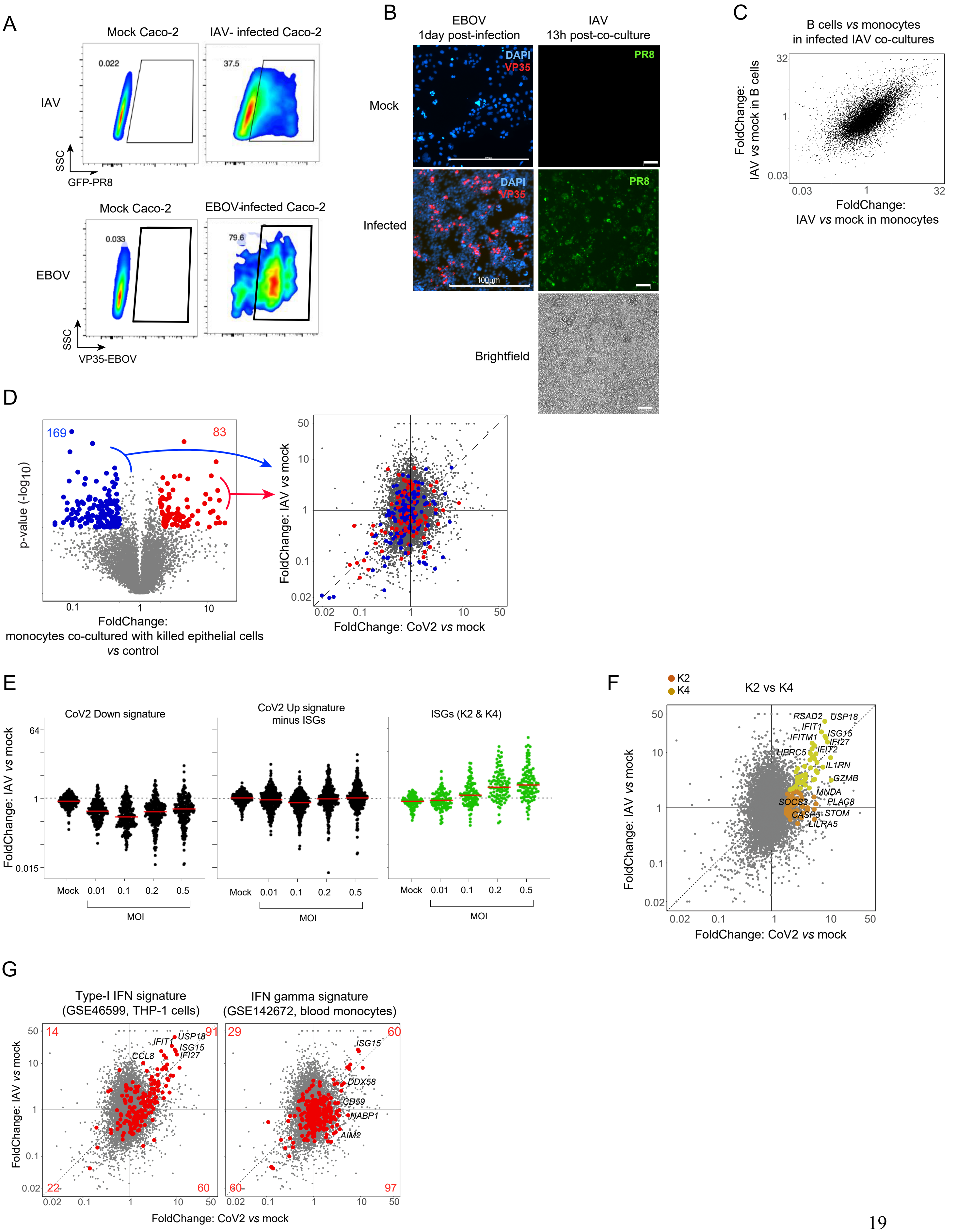
A
Mock Caco-2
IAV- infected Caco-2
IAV
0.022
37.5
SSC
GFP-PR8
Mock Caco-2
EBOV-infected Caco-2
EBOV
0.033
79.6
SSC
VP35-EBOV
B
EBOV
1day post-infection
IAV
13h post-co-culture
Mock
Infected
Brightfield
DAPI
VP35
PR8
100µm
C
B cells vs monocytes
in infected IAV co-cultures
FoldChange:
IAV vs mock in B cells
FoldChange:
IAV vs mock in monocytes
32
1
0.03
D
169
83
p-value (-log10)
0.1
1
10
FoldChange:
monocytes co-cultured with killed epithelial cells
vs control
FoldChange: IAV vs mock
FoldChange: CoV2 vs mock
50
10
1
0.1
0.02
E
CoV2 Down signature
CoV2 Up signature
minus ISGs
ISGs (K2 & K4)
FoldChange: IAV vs mock
64
1
0.015
Mock
0.01
0.1
0.2
0.5
MOI
F
K2
K4
K2 vs K4
RSAD2
USP18
IFIT1
IFITM1
ISG15
IFI27
HERC5
IFIT2
IL1RN
GZMB
MNDA
SOCS3
PLAC8
CASP5
STOM
LILRA5
FoldChange: IAV vs mock
FoldChange: CoV2 vs mock
G
Type-I IFN signature
(GSE46599, THP-1 cells)
IFN gamma signature
(GSE142672, blood monocytes)
14
91
22
60
IFIT1
USP18
ISG15
IFI27
CCL8
29
60
60
97
ISG15
DDX58
CD59
NABP1
AIM2
FoldChange: IAV vs mock
FoldChange: CoV2 vs mock

Figure S3

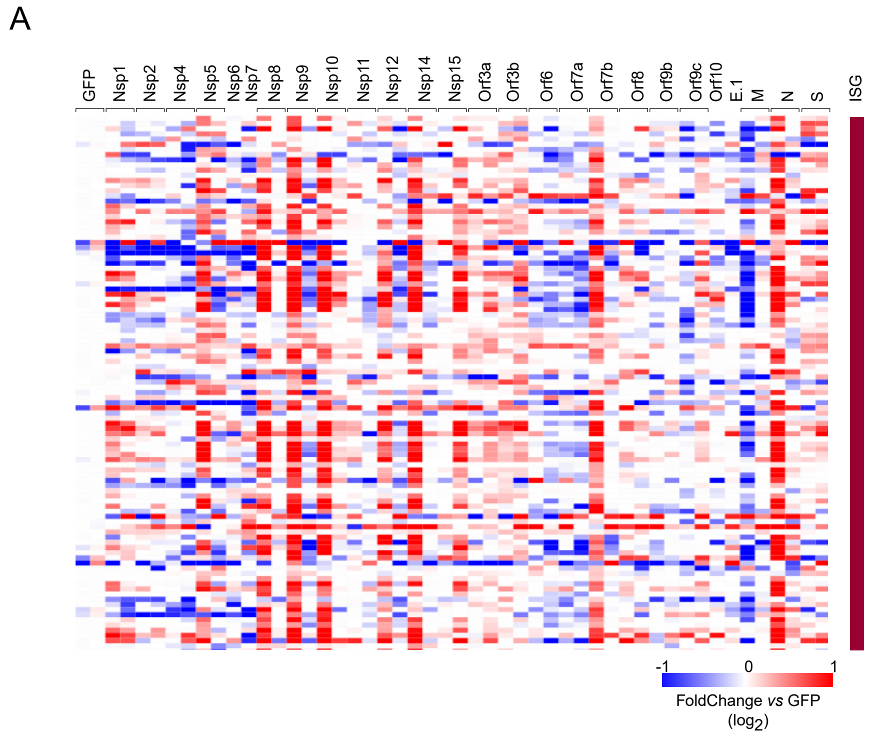

A
GFP
Nsp1
Nsp2
Nsp4
Nsp5
Nsp6
Nsp7
Nsp8
Nsp9
Nsp10
Nsp11
Nsp12
Nsp14
Nsp15
Orf3a
Orf3b
Orf6
Orf7a
Orf7b
Orf8
Orf9b
Orf9c
Orf10
E.1
M
N
S
ISG
-1
0
1
FoldChange vs GFP (log2)

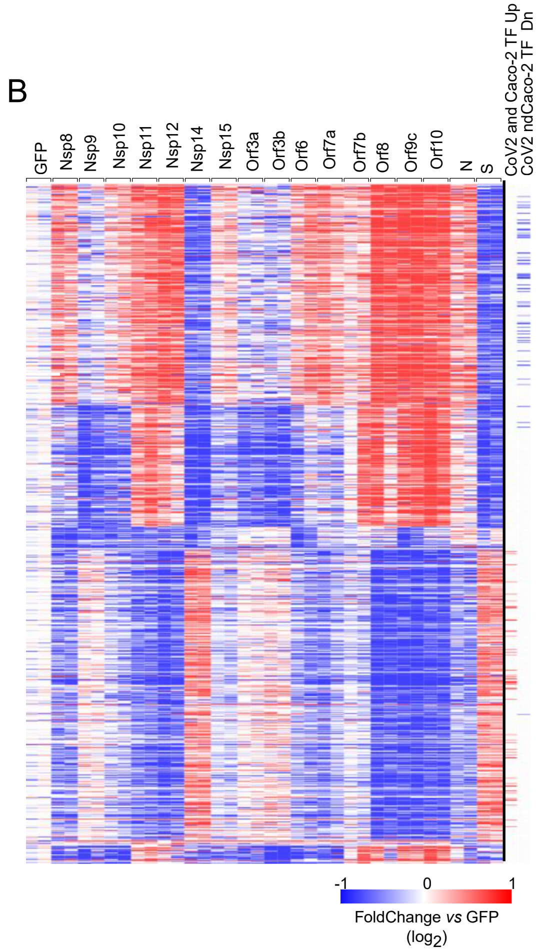

B
GFP
Nsp8
Nsp9
Nsp10
Nsp11
Nsp12
Nsp14
Nsp15
Orf3a
Orf3b
Orf6
Orf7a
Orf7b
Orf8
Orf9c
Orf10
N
S
CoV2 and Caco-2 TF Up
CoV2 ndCaco-2 TF Dn
-1
0
1
FoldChange vs GFP (log2)

Figure S4

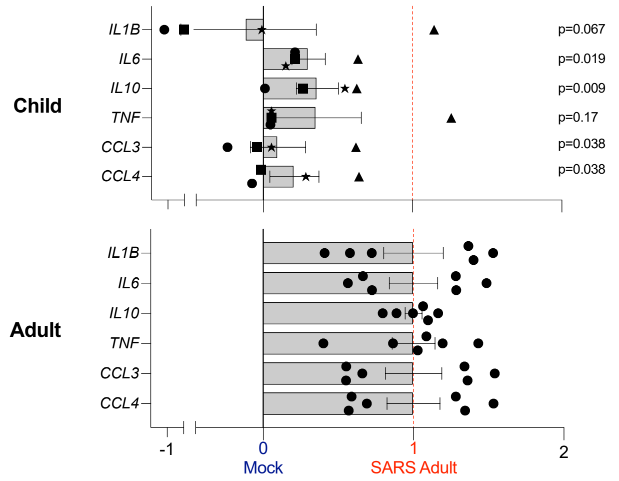

Child
IL1B
IL6
IL10
TNF
CCL3
CCL4
p=0.067
p=0.019
p=0.009
p=0.17
p=0.038
p=0.038
Adult
IL1B
IL6
IL10
TNF
CCL3
CCL4
-1
0
1
2
Mock
SARS Adult

SI DATASET LEGENDS

Dataset S1: Cov2 signature

Dataset S2: Gene ontology analyses of the CoV2 signature

Dataset S3: Donor information and viral reads among the different samples and conditions

Dataset S4: Heatmap of the CoV2 signature among all CD14+ samples (Fig. 2I - FoldChange vs the average of the respective mocks)
